# Supplementary material for: Conserved structures of neural activity in sensorimotor cortex of freely moving rats allow cross-subject decoding
Source: Nat Commun. 2022 Dec 2;13:7420. doi: 10.1038/s41467-022-35115-6 (PMC9715555; doi:10.1038/s41467-022-35115-6)
Supplement: Supplementary file 1 — Supplementary Information [file 41467_2022_35115_MOESM1_ESM.pdf]

Conserved structures of neural activity in sensorimotor cortex of freely moving rats allow cross-subject decoding

Supplementary Information

Svenja Melbaum[1,2*], Eleonora Russo[3,4*], David Eriksson[2,5],
Artur Schneider[2,5], Daniel Durstewitz[4], Thomas Brox[1,2], Ilka Diester[2,5,6**]

[1]Computer Vision Group, Dept. of Computer Science,
University of Freiburg, 79110 Freiburg, Germany.
[2]IMBIT//BrainLinks-BrainTools,
University of Freiburg, Georges-Köhler-Allee 201, 79110 Freiburg, Germany.
[3]Department of Psychiatry and Psychotherapy, University Medical Center,
Johannes Gutenberg University, 55131 Mainz, Germany.
[4]Department of Theoretical Neuroscience, Central Institute of Mental Health,
Medical Faculty Mannheim, University of Heidelberg, 68159 Mannheim, Germany.
[5]Optophysiology Lab, Faculty of Biology,
University of Freiburg, 79110 Freiburg, Germany.
[6]Bernstein Center Freiburg, University of Freiburg, 79104 Freiburg, Germany.

[*]These authors contributed equally
[**]Corresponding author: ilka.diester@biologie.uni-freiburg.de

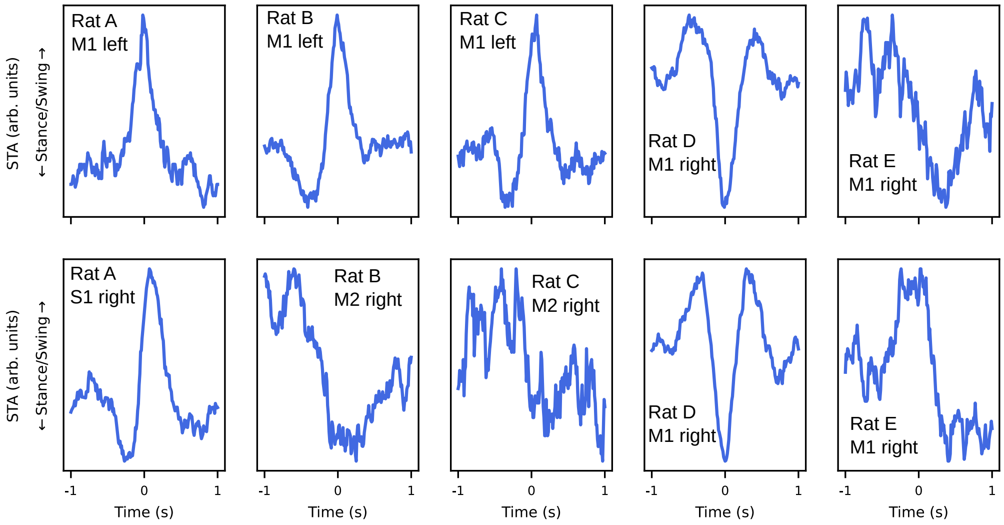

Supplementary Fig. 1: **Significantly coupled neurons showed clear peaks in the STAPSSS.** STAPSSS for the right front paw of 10 example neurons from different motor areas from five sessions of different rats. Extends Fig. 1d from the main paper. Source data are provided as a Source Data file.

Supplementary Table 1: **Statistics of the recording sessions**. Dates of implantations and the recording periods for each animal.

	Rat A	Rat B	Rat C	Rat D	Rat E	Rat F
Implantation	20/04/2017	19/04/2017	27/04/2017	11/04/2017	25/04/2017	01/01/2017
First recording	01/06/2017	01/06/2017	01/06/2017	01/06/2017	01/06/2017	07/06/2017
Last recording	15/08/2017	21/08/2017	08/07/2017	21/08/2017	25/08/2017	22/08/2017

Supplementary Table 2: **ANOVA results for paw coupling**. Paw coupling was defined as the ratio between the STAPSSS standard deviation and the control standard deviation (see main text). Three-way ANOVAs were calculated separately for each paw on all recorded neurons ($n = 3,723$, main effects area, hemisphere, rat; interaction effect area and hemisphere). The table contains the corresponding F and p values.

Paw	Area	Hemisphere	Area x Hemisphere	Rat
Right front	$66.8, p = 3.26\text{e}{-29}$	$108.9, p = 3.89\text{e}{-25}$	$18.2, p = 1.31\text{e}{-8}$	$28.6, p = 1.51\text{e}{-28}$
Left front	$41.6, p = 1.34\text{e}{-18}$	$17.2, p = 3.53\text{e}{-5}$	$2.2, p = 0.10$	$37.8, p = 5.83\text{e}{-38}$
Right hind	$25.7, p = 8.01\text{e}{-12}$	$4.2, p = 0.041$	$5.6, p = 0.003$	$23.6, p = 1.85\text{e}{-23}$
Left hind	$67.5, p = 1.65\text{e}{-29}$	$6.4, p = 0.01$	$3.82, p = 0.02$	$13.5, p = 4.22\text{e}{-13}$

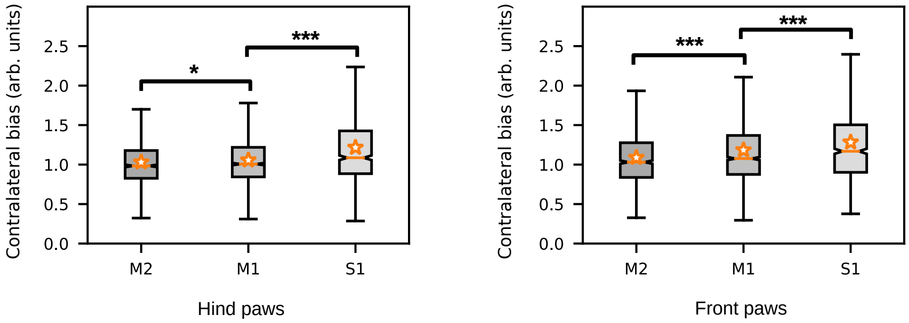

Supplementary Fig. 2: **Contralateral bias was largest in S1.** Contralateral bias for the front and hind paws per area, averaged over neurons ($n = 1169, n = 1692, n = 862$ for M2, M1, S1). The bias increased from anterior to posterior regions for both the front and hind paws. Stars denote the results of the post-hoc Tukey–Kramer tests. The boxplots show the median and the first and third quartile, the whiskers extend to 1.5 times the interquartile range. Orange stars denote mean values, and notches denote the 95% confidence intervals for the median. See the main text for definitions of paw coupling and bias. $*p < 0.05, ***p < 0.001$. The precise p values are .042 for M2 and M1 and $p = 1.99\text{e}{-17}$ for M1 and S1 for the hind paws, and $p = 1.73\text{e}{-8}$ for M2 and M1 and $p = 6.09\text{e}{-6}$ for M1 and S1 for the front paws. Source data are provided as a Source Data file.

Supplementary Table 3: **Tukey–Kramer post-hoc results for the paw coupling**. Paw coupling was defined as the ratio between the STAPSSS standard deviation and the control standard deviation (see main text). After calculating three-way ANOVAs separately for each paw on all recorded neurons ($n = 3,723$, main effects area, hemisphere, rat; interaction effect area and hemisphere). post-hoc Tukey–Kramer tests were performed to assess pairwise differences between areas within one hemisphere. The table contains the corresponding p values. This table provides background data for Fig. 1e.

Front left	Front right	Hind left	Hind right
M2 M1 left, $p = .028$	M2 M1 left, $p = 3.61\text{e}{-13}$	M2 M1 left, $p = 3.45\text{e}{-5}$	M2 M1 left, $p = 1.75\text{e}{-4}$
M2 S1 left, $p = 2.15\text{e}{-5}$	M1 S1 left, $p = 2.15\text{e}{-9}$	M1 S1 left, $p = .0028$	M1 S1 left, $p = .0024$
S1 M1 right, $p = 8.76\text{e}{-4}$	S1 M2 right, $p = .0012$	S1 M1 right, $p = 8.16\text{e}{-22}$	S1 M1 right, $p = .0019$
M1 M2 right, $p = 7.38\text{e}{-6}$	M2 M1 right, $p = .006$	S1 M2 right, $p = 1.40\text{e}{-20}$	S1 M2 right, $p = .030$

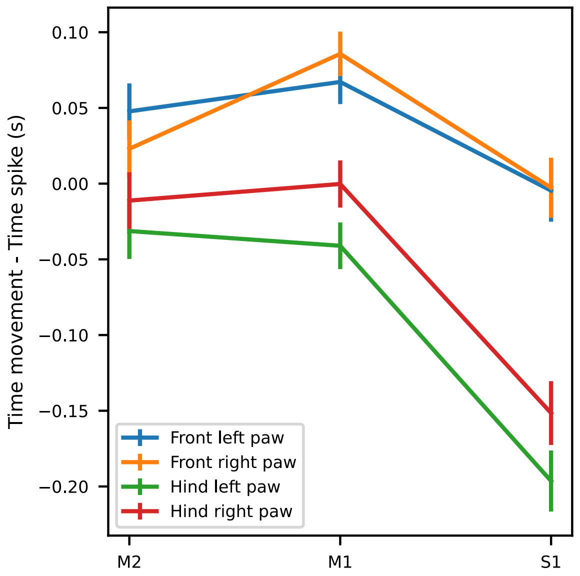

Supplementary Fig. 3: **Temporal relationship between movement and brain-area-specific neuronal activity in M2, M1, and S1.** Movement refers here to the STAPSSS peak. Negative values indicate that the spikes followed the movement (in the form of the STAPSSS peak); positive values indicate that the spikes preceded the movement. The spikes in S1 tended to occur after movements, significantly later than the spikes in M2 and M1. The mean and standard error of the mean over all neurons in each area are shown ($n = 1169, n = 1692, n = 862$ for M2, M1, S1). Refers to the main paper's Fig. 1. Source data are provided as a Source Data file.

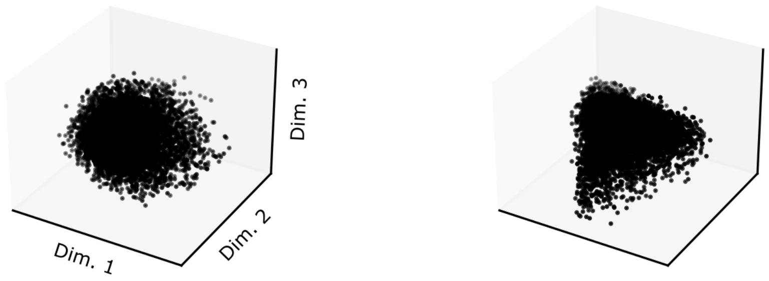

Supplementary Fig. 4: **Some population structures did not show any apparent structure.** Two example sessions (from Rats A and B, respectively) with random-like, low-dimensional neural projections. Refers to the main paper's Fig. 2a. Source data are provided as a Source Data file.

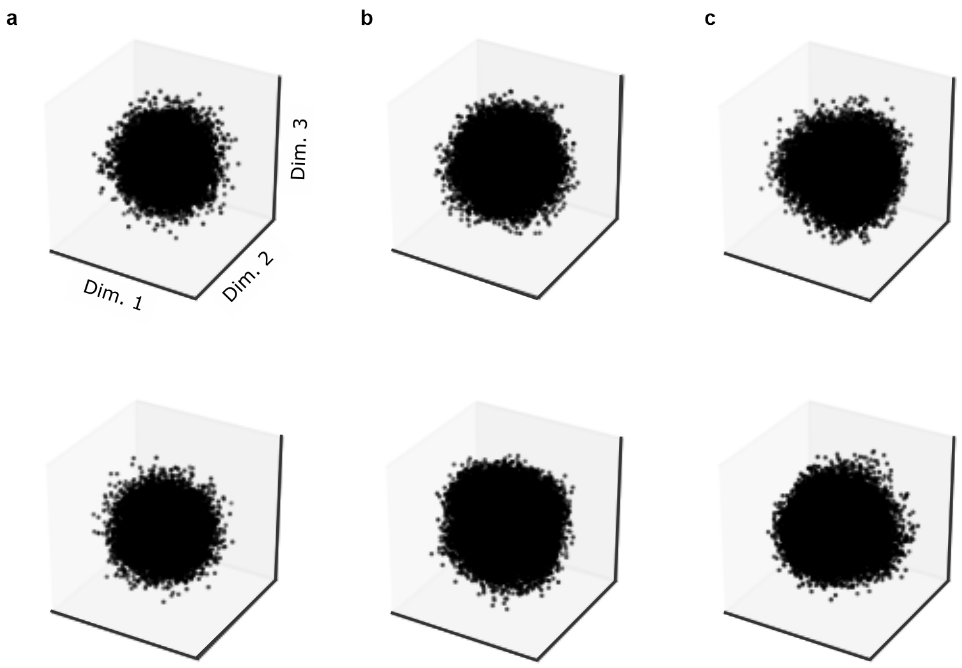

Supplementary Fig. 5: **Control dimensionality reductions with shuffled neuronal activity did not show any apparent structure.** LEM projections for neuron-shuffled (a), time-shuffled (b), and time-shifted (c) data for one session of Rat A (upper row) and Rat B (lower row). For neuron shuffling, units were permuted randomly for each time point. For time shuffling, time points were permuted randomly for each neuron. For time shifting, the spike trains of the neurons were randomly shifted against each other. The two sessions are the same as in Fig. 2b of the main paper. Source data are provided as a Source Data file.

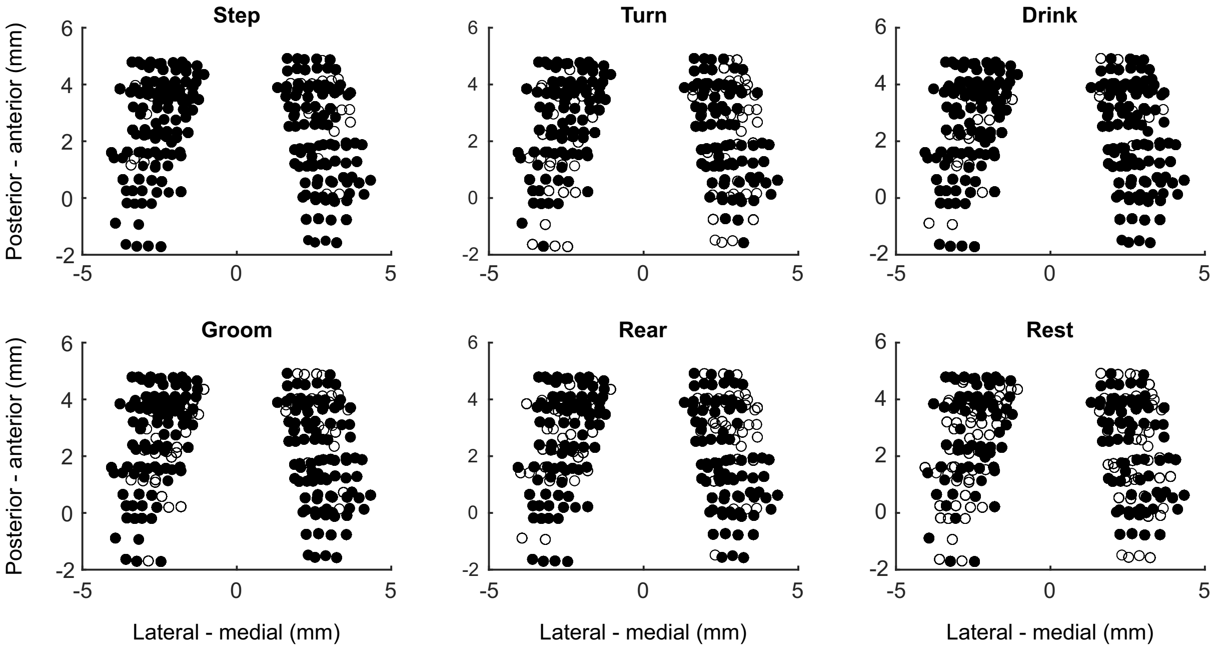

Supplementary Fig. 6: **Encoding of different behaviours across the sensorimotor cortex.** Recording sites of the single-units which were significantly more active during each of the six behaviors. The black dots mark recording sites with significant units.

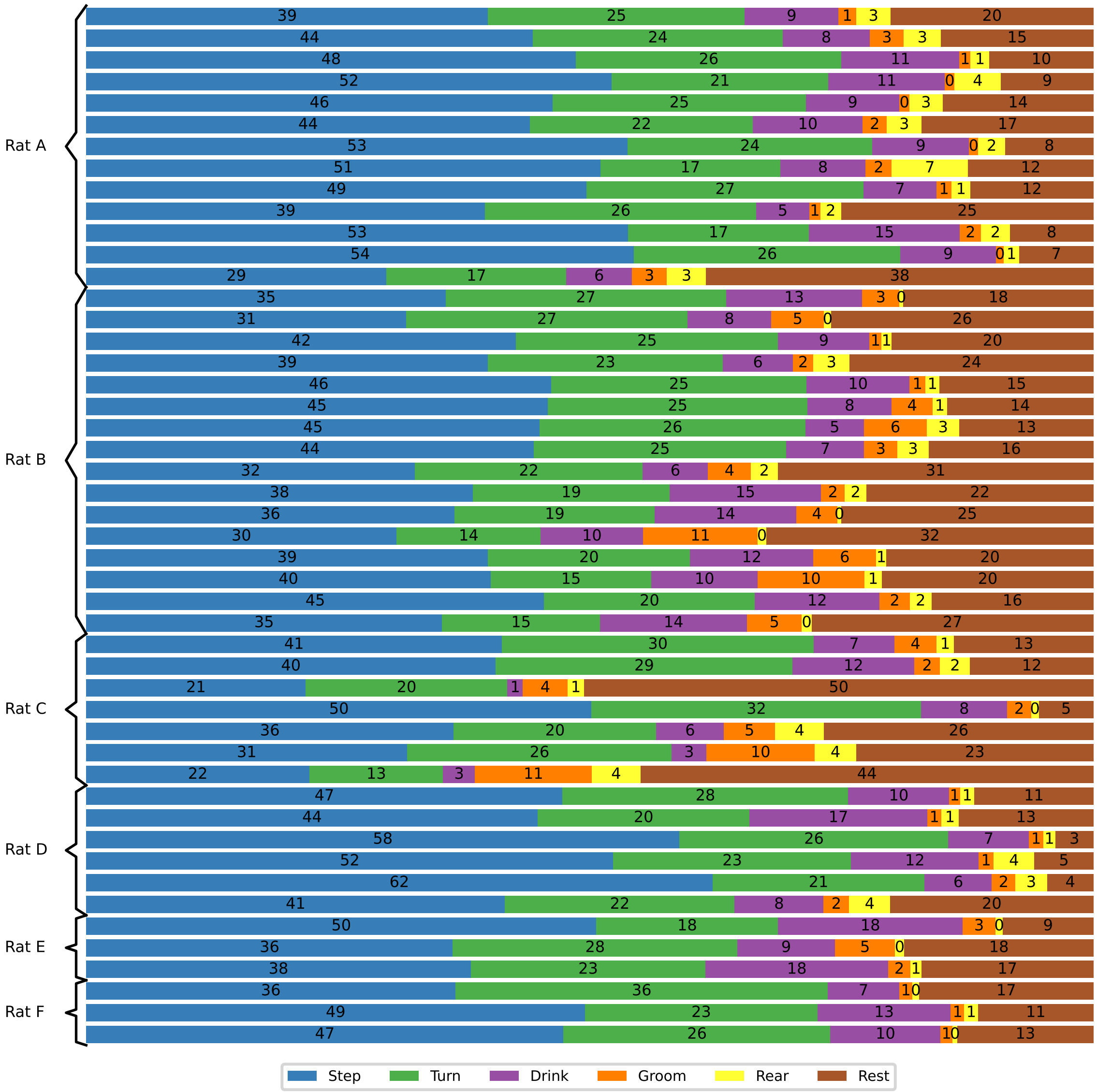

Supplementary Fig. 7: **Histograms of behavior illustrating the distribution of behavioral classes.** One row reflects one session. Provides background for Fig. 2 in the main paper.

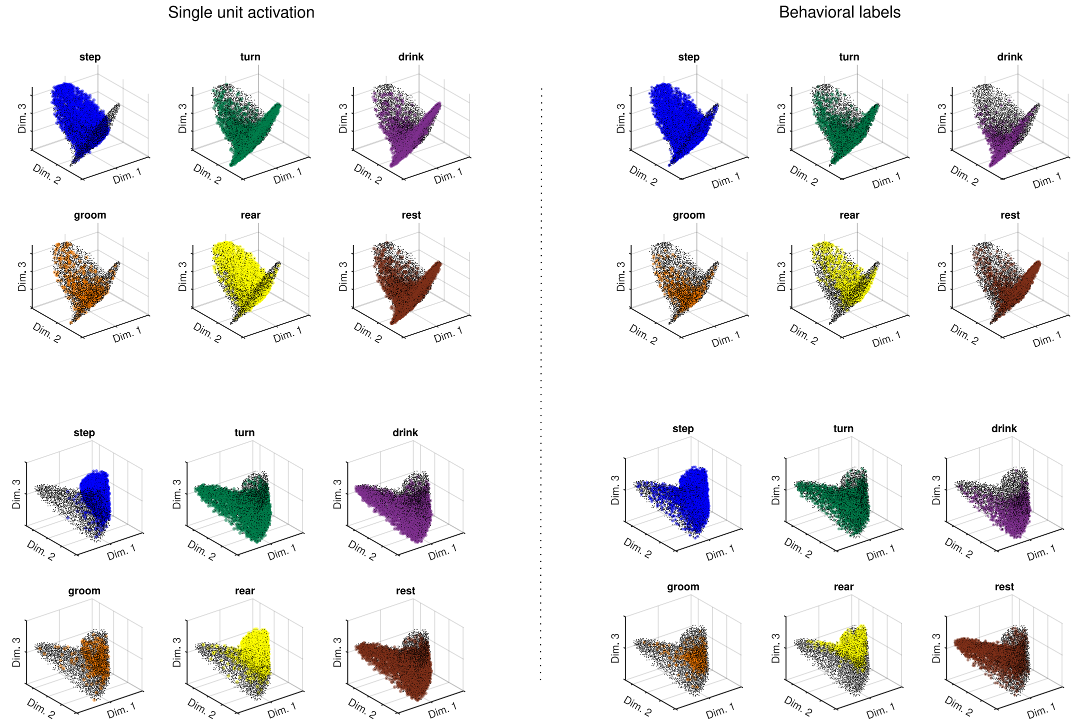

Supplementary Fig. 8: **Comparison of single-unit firing patterns and localization of behaviors in the LEM space.** (Left) The LEM manifold from one example session of Rat A is color-coded according to the firing of six different example single-units significantly responding to the six behavioral classes. Colored dots mark time points when the unit fired above its 75th empirical quartile, while black dots mark any other time point. (Right) On the same LEM manifold as in the left panel, colored dots mark time points corresponding to the six behavioral labels. Different orientations of the same manifold are shown from top to bottom. Source data are provided as a Source Data file.

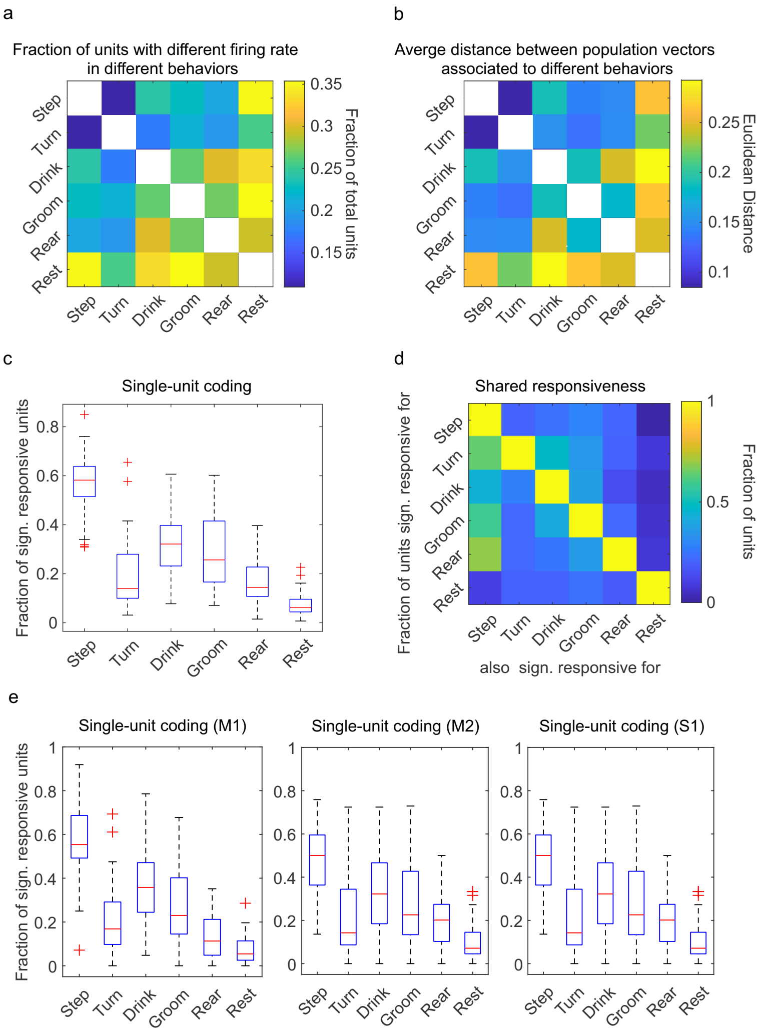

Supplementary Fig. 9: **Single-unit coding of different behaviors.** (a) Fraction of single-units changing their average firing rate when active during different behaviors as the fraction of significant post-hoc comparisons ($\alpha = 0.05$) of a Kruskal–Wallis test on the firing rate of single-units during the six identified behaviors. Differences in sample size across classes were compensated with down-sampling (see Methods). (b) Average distance between the population vectors of the LEM space (dim = 10) associated with different behaviors. (c) Fraction of units significantly more active during each of the behaviors (two-sided Wilcoxon rank-sum test, with Benjamini–Hochberg correction for multiple comparisons, $\alpha = 0.05$, $n = 48$ for all behavioral labels). The median (red line) across sessions, the 25th and 75th percentiles (blue), the most extreme data points (whiskers), and outliers (crosses) are shown. (d) Based on (c), the fraction of units with shared responsiveness to multiple behaviors. (e) Same as (c) but computed selectively on the single-units recorded in M1, M2, and S1 ($n = 48$ for all behavioral labels and regions). Source data are provided as a Source Data file.

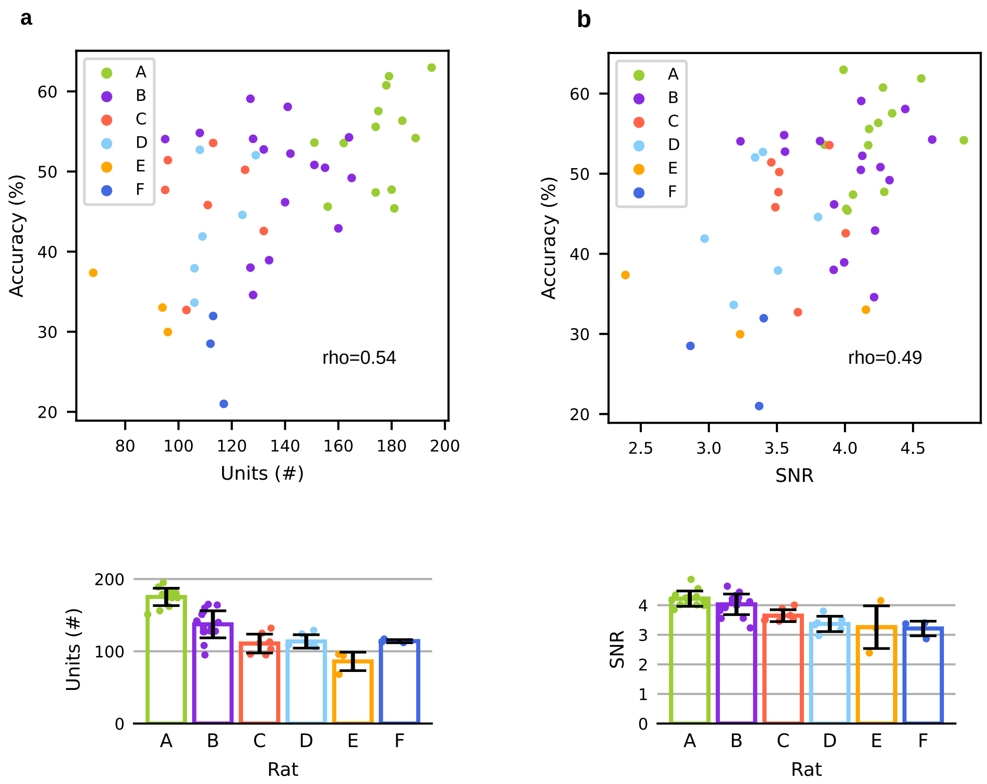

Supplementary Fig. 10: **Correlation between accuracies and units/SNR.** (a) Top: Accuracies versus the number of units per session for the six rats. Bottom: Average number of units per rat and error bars for the standard deviation across sessions. (b) Top: Accuracies versus the mean signal-to-noise ratio (SNR) per session for the six rats. Bottom: Average SNR per rat, with error bars for the standard deviation. The bottom panels are the same as in Fig. 5d in the main paper, but with Rat F included. Number of sessions $n = 13, 16, 7, 6, 3, 3$ for rats A-F. Refers to Fig. 2. Source data are provided as a Source Data file.

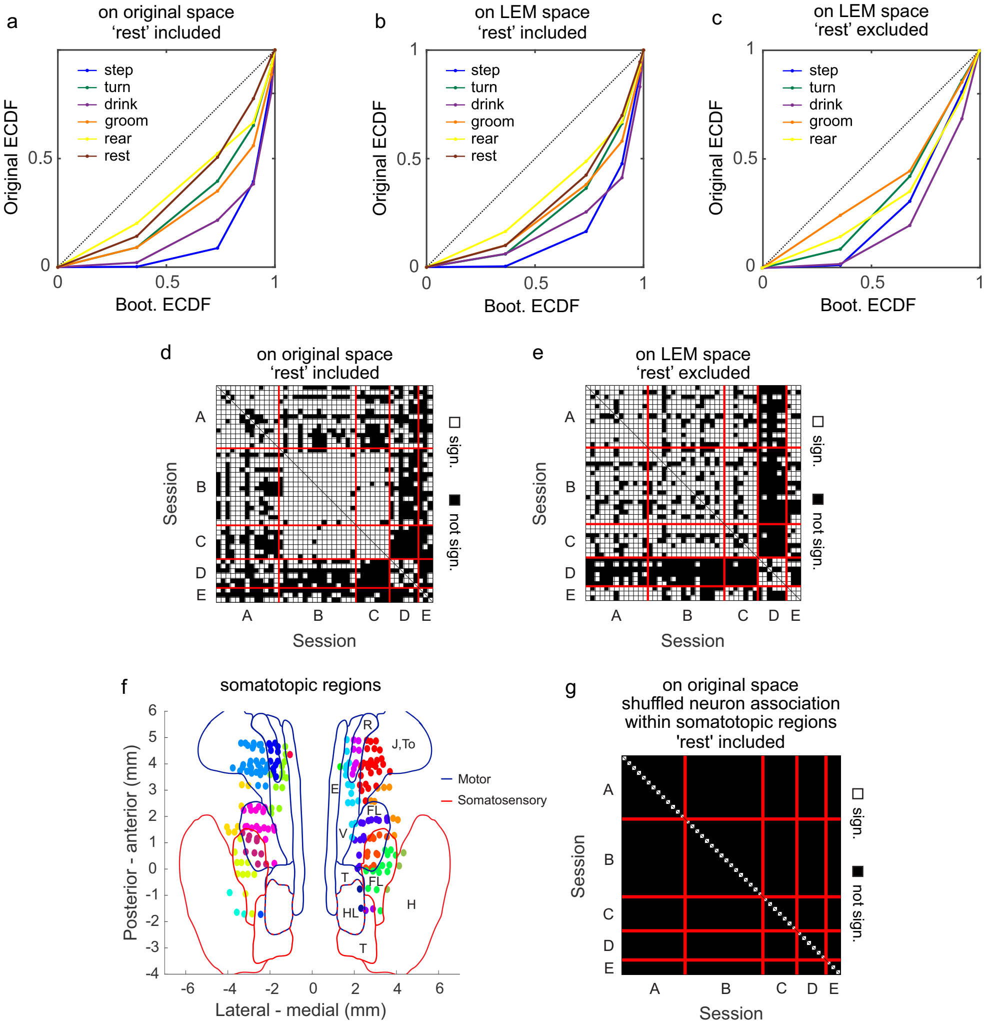

Supplementary Fig. 11: **Similarity among the polytopes of different sessions.** (Continued on the following page.)

Supplementary Fig. 11: **Similarity among the polytopes of different sessions.** (a–c) Probability–probability (p–p) plot comparing the original and bootstrapped empirical cumulative distribution function (ECDF) of the statistic s_i^{vw}, which compares the ranked distances between the polytope vertexes across sessions (see Methods for a formal definition). The ECDFs of s_i^{vw} were computed for each behavioral class i (color-coded) on the original recordings (a), on the 20-dimensional LEM space (b), and on the 20-dimensional LEM space with the class "rest" excluded from the test (c). In a p–p plot, equal distributions overlap with the diagonal (dotted line). (d–e) are the same as in Fig. 3b, but with computing distances on the rate vectors of the original recording space (d) and on the 20-dimensional LEM space with the "rest" class excluded from the test (e). Of the 990 possible session pairs, 60% and 61% in (d) and (e), respectively, had a p-value below 0.05 (one-sided bootstrap test, no correction applied for multiple comparisons). (f) Somatotopic regions adapted from [1, 2] for the sensory (red) and motor (blue) control of the eye (E), rhinarium (R), vibrissa (V), hindlimb (HL), forelimb (FL), trunk (T), jaw (J), and tongue (To). Circles mark the electrode's positions. (g) Same as (d), but with computing distances on population vectors built by shuffling the identities of single neurons recorded within the same somatotopic region 500 times, to remove the relation between the correlation structure within somatotopic regions while maintaining this structure across regions. Marked in black or white are session comparisons with average p-values above or below 0.05, respectively. The cross-correlation structure derived from the presence of somatotopic organization in the sensorimotor cortex does not guarantee polytope similarity. Source data are provided as a Source Data file.

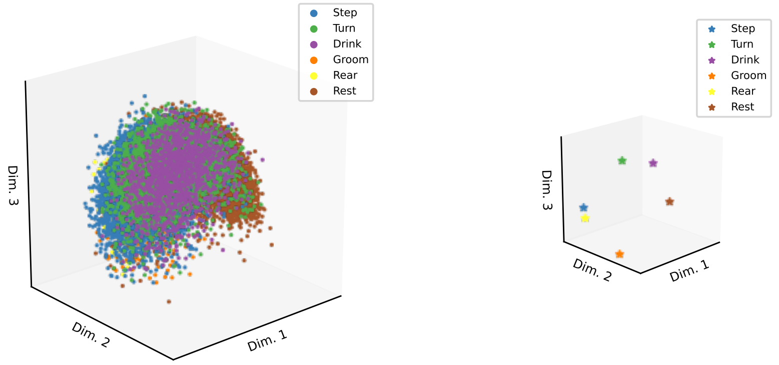

Supplementary Fig. 12: **Population structure in the Isomap space.** Left: All points. Right: For better visualization, only the averages of the six behavioral classes were plotted. One session of Rat A is shown here. Source data are provided as a Source Data file.

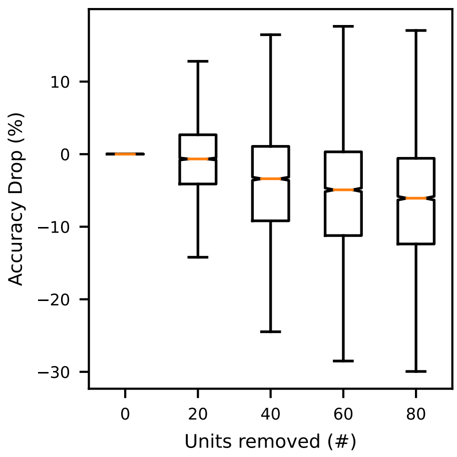

Supplementary Fig. 13: **Generalization worsened with fewer units.** We repeated the generalization experiment from Fig. 5a for all sessions with a generalization accuracy of at least 55% (19 sessions). The accuracy decreased for LEM structures that were computed after removing 20, 40, 60, or 80 units from each session compared to the accuracies with the full number of units. Thus, accuracy decreased with fewer units. The boxplots show the median (with notches for the 95% confidence interval) and the first and third quartile, the whiskers extend to 1.5 times the interquartile range of the accuracies ($n = 5415$ for 15 experiments per 19 $\times$ 19 session pairings). Source data are provided as a Source Data file.

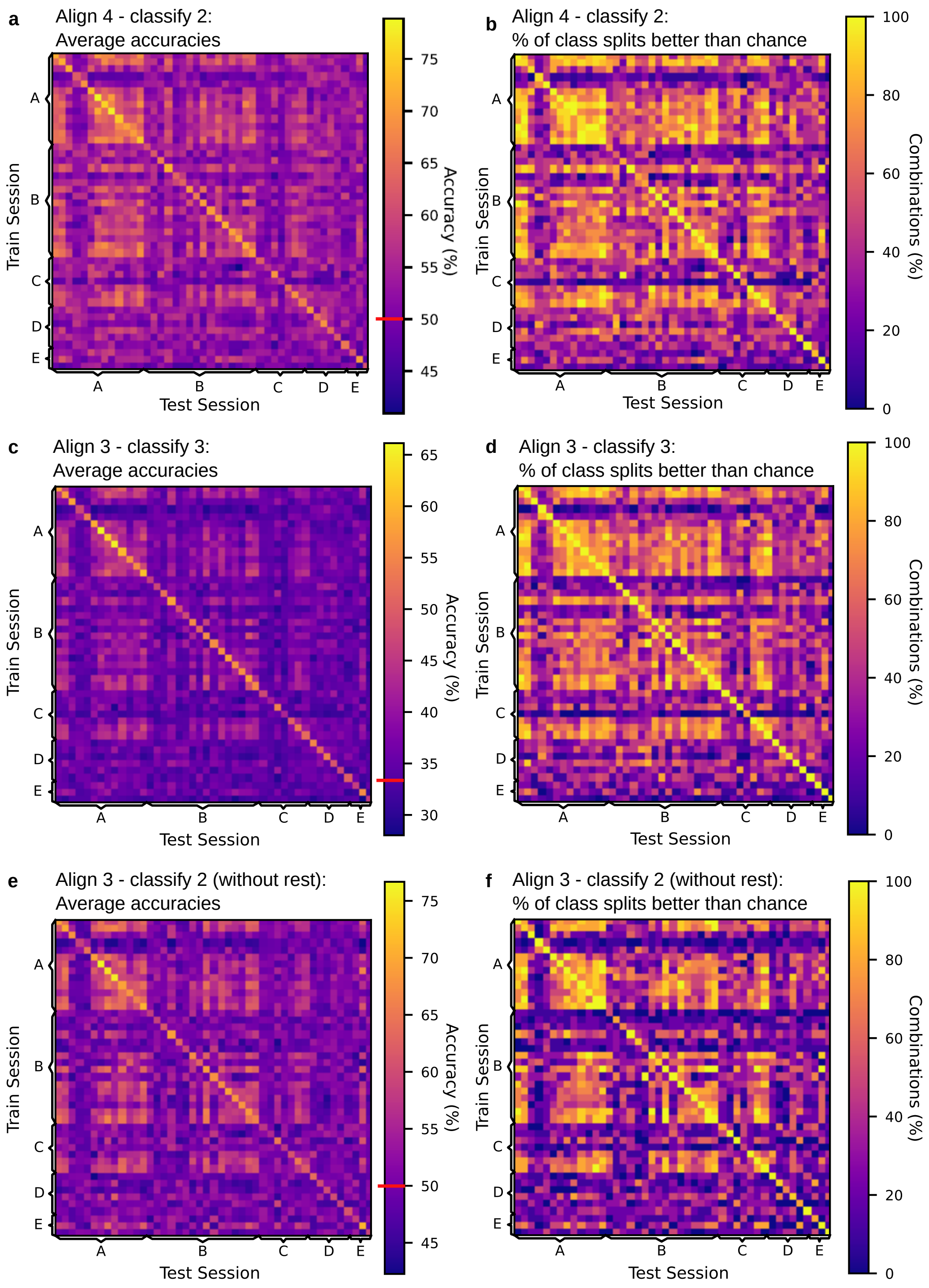

Supplementary Fig. 14: **Further cross-subject and cross-session generalization studies.** (Continued on the following page.)

Supplementary Fig. 14: **Further cross-subject generalization experiments.** For all plots, training and test data on the diagonal originated from the same session. Off-diagonal entries show testing on data other than the training session. (a) Mean per-class accuracies across training and test sessions when aligning on four and testing on two classes. The chance level was 50% (red line). Same plot of Fig. 5a reported here for comparison with panels (b,c,e) (b) Percentage of splits (out of 15) of the six behavioral classes into the alignment and decoding sets with significantly higher mean per-class decoding accuracy than chance (50%). Significance was calculated over 20 training runs at .05 significance level with Bonferroni correction using a one-tailed sign test. The accuracy was significantly better than chance in $47.55\%(14445/(15*45*45) = 14445/30375)$ of the experiments. (c–f) We conducted further generalization experiments with a more difficult setting (align on three classes and classify three classes, c–d; experiment without the "rest" class, e–f). (c) Mean per-class accuracies across training and test sessions when aligning on three and testing on three classes. The chance level was 33.33%. Values were averaged over 20 runs and 20 possible splits of the six behavioral classes into alignment/decoding sets. (d) Percentage of splits with above-chance per-class decoding accuracy, as in (b), but by aligning on three classes and testing with three classes, with 20 combinations total and a chance level of 33.33% (red line). In $44.28\%(17934/40500)$ of the experiments, the accuracy was significantly better than chance. (e) Mean per-class accuracies across training and test sessions when aligning on three and testing on two classes, without the "rest" class. The chance level was 50% (red line). Values were averaged over 20 runs and 10 possible splits of the five behavioral classes into alignment/decoding sets. (f) This is the same as in (b) and (d) for aligning on three and testing on two classes without the class "rest," with 10 combinations in total and a chance level of 50%. In $37.25\%(7545/20250)$ of the experiments, the accuracy was significantly better than chance. Source data are provided as a Source Data file.

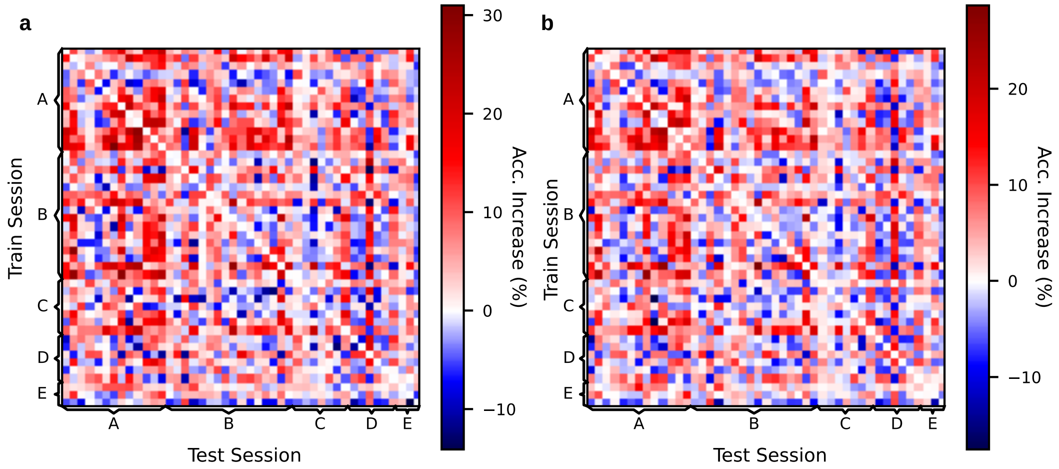

Supplementary Fig. 15: **Decoding accuracy gain through neural manifold alignment.** Accuracy gains for aligned versus unaligned neural structures for the decoding of (a) two classes and (b) three classes. Values are averaged over all class combinations. In most cases, the accuracies were higher after alignment (red color spectrum) by up to 20–30%. Refers to Fig. 5a in the main paper and Supplementary Fig. 14a–d.

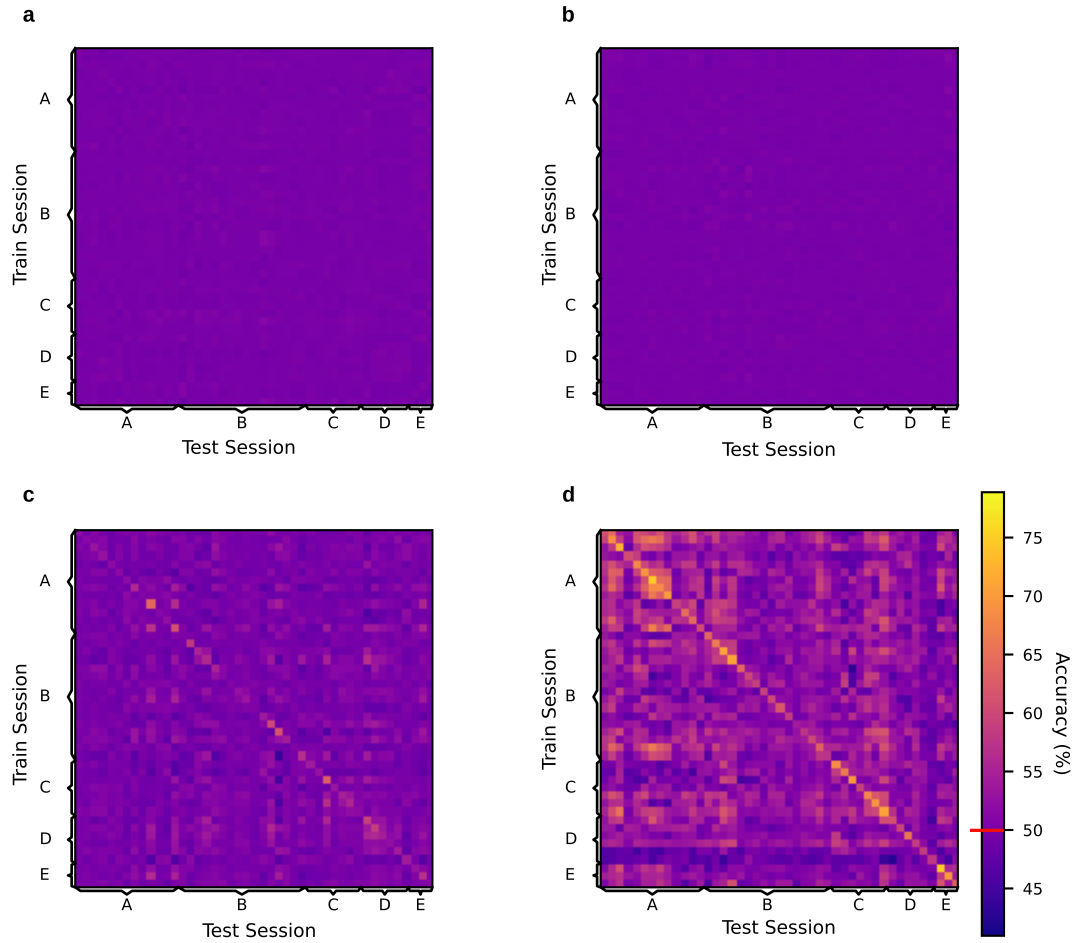

Supplementary Fig. 16: **Control generalization experiments.** Generalization results on neuron-shuffled (a), time-shuffled (b), and time-shifted (c) data, as well as the LEM space from non-binarized spikes (d). Mean per-class accuracies across training and test sessions when aligning on four and testing on two classes (chance level of 50%, red line) are shown, as in Fig. 5a.

Supplementary References

[1] Hall, R. D. & Lindholm, E. P. Organization of motor and somatosensory neocortex in the albino rat. *Brain research* **66**, 23–38 (1974).

[2] Neafsey, E. J. & Sievert, C. A second forelimb motor area exists in rat frontal cortex. *Brain research* **232**, 151–156 (1982).
