# Supplementary material for: Conserved structures of neural activity in sensorimotor cortex of freely moving rats allow cross-subject decoding
Source: Nat Commun. 2022 Dec 2;13:7420. doi: 10.1038/s41467-022-35115-6 (PMC9715555; doi:10.1038/s41467-022-35115-6)
Supplement: Supplementary file 2 — Reporting Summary [file 41467_2022_35115_MOESM2_ESM.pdf]

nature portfolio

Corresponding author(s): Ilka Diester
Last updated by author(s): 11/11/2022

Reporting Summary

Nature Portfolio wishes to improve the reproducibility of the work that we publish. This form provides structure for consistency and transparency in reporting. For further information on Nature Portfolio policies, see our Editorial Policies and the Editorial Policy Checklist.

Statistics

For all statistical analyses, confirm that the following items are present in the figure legend, table legend, main text, or Methods section.

n/a	Confirmed	
☐	☒	The exact sample size (n) for each experimental group/condition, given as a discrete number and unit of measurement
☐	☒	A statement on whether measurements were taken from distinct samples or whether the same sample was measured repeatedly
☐	☒	The statistical test(s) used AND whether they are one- or two-sided<br>*Only common tests should be described solely by name; describe more complex techniques in the Methods section.*
☒	☐	A description of all covariates tested
☐	☒	A description of any assumptions or corrections, such as tests of normality and adjustment for multiple comparisons
☐	☒	A full description of the statistical parameters including central tendency (e.g. means) or other basic estimates (e.g. regression coefficient) AND variation (e.g. standard deviation) or associated estimates of uncertainty (e.g. confidence intervals)
☐	☒	For null hypothesis testing, the test statistic (e.g. F, t, r) with confidence intervals, effect sizes, degrees of freedom and P value noted<br>*Give P values as exact values whenever suitable.*
☒	☐	For Bayesian analysis, information on the choice of priors and Markov chain Monte Carlo settings
☒	☐	For hierarchical and complex designs, identification of the appropriate level for tests and full reporting of outcomes
☐	☒	Estimates of effect sizes (e.g. Cohen's d, Pearson's r), indicating how they were calculated

Our web collection on statistics for biologists contains articles on many of the points above.

Software and code

Policy information about availability of computer code

Data collection	Data was collected using the INTAN USB evaluation board (https://intantech.com/RHD_USB_interface_board.html).
Data analysis	The code for the principal functions created for this manuscript is available at https://doi.org/10.5281/zenodo.7296960.<br>The following software was used:<br>Matlab==2019a;<br>Python==3.6;<br>pandas==0.24.1;<br>matplotlib==3.0.0;<br>numpy==1.14.5;<br>scikit-learn==0.20.0;<br>scipy==1.1.0;<br>tensorflow==1.10.0.

For manuscripts utilizing custom algorithms or software that are central to the research but not yet described in published literature, software must be made available to editors and reviewers. We strongly encourage code deposition in a community repository (e.g. GitHub). See the Nature Portfolio guidelines for submitting code & software for further information.

Data

Policy information about availability of data

All manuscripts must include a data availability statement. This statement should provide the following information, where applicable:

- Accession codes, unique identifiers, or web links for publicly available datasets
- A description of any restrictions on data availability
- For clinical datasets or third party data, please ensure that the statement adheres to our policy

The data that support the findings of this study are available from the corresponding authors upon reasonable request
Example dataset are available under https://github.com/Optophys/Conserved_structures_cortex

Field-specific reporting

Please select the one below that is the best fit for your research. If you are not sure, read the appropriate sections before making your selection.

☒ Life sciences ☐ Behavioural & social sciences ☐ Ecological, evolutionary & environmental sciences

For a reference copy of the document with all sections, see nature.com/documents/nr-reporting-summary-flat.pdf

Life sciences study design

All studies must disclose on these points even when the disclosure is negative.

Sample size	The statistical power of the novel randomized behavior used here could not be known in before hand. Therefore we performed daily recordings of six rats until we saw a decline in the signal quality. This provided enough data for statistical inference after correction for data dependence.
Data exclusions	Only sessions were considered with > 30 m duration. (106 sessions left)<br>- Stance/swing decoding: 104 sessions left due to insufficient data for train\test split<br>- LEM: Only time points with at least 15 active units were retained. Since we restricted further analysis to sessions with at least 5000 valid time points, we considered only 95 of the 106 sessions.<br>- Behavioral labeling: The 48 sessions were chosen based on them having a clear saddle-like shape in the LEM population structure and at least five significantly paw-coupled units according to our STAPSSS analysis as a proxy for high recording quality. Behavioural labels were extracted from video recordings. In the rare cases the rat was not visible because located near the borders of the arena, the samples were excluded from labeling. This exclusion criterion was pre-established.<br>- Cross-subject decoding: 3 sessions from rat F were excluded due to poor recording quality
Replication	We recorded from 6 animals and, in average, we performed 10-20 sessions per animal. The behavioral repetitivity across sessions was minimal, therefore each session could be seen as an behaviorally independent sample. The neuronal activity across the sessions was not independent since a single neuron could be recorded for multiple days. For data analysis time-shifted, time-shuffled, and identity-shuffled controls were performed. We found that the quality of cross-subject decoding depended on the number of neurons recorded in each session. For each combination of pairs of recorded animals, we could find at least two sessions in which the recorded manifold showed above chance similarity across animals. Among all possible cross-session classification tests, 48% where significantly above chance.
Randomization	The randomization algorithm was identical for all animals and newly seeded for every session and animal.
Blinding	The behavioral training, reward control, and recordings were autonomously done by a computer whose program was the same for all animals.

Reporting for specific materials, systems and methods

We require information from authors about some types of materials, experimental systems and methods used in many studies. Here, indicate whether each material, system or method listed is relevant to your study. If you are not sure if a list item applies to your research, read the appropriate section before selecting a response.

Materials & experimental systems

n/a	Involved in the study
☒	☐ Antibodies
☒	☐ Eukaryotic cell lines
☒	☐ Palaeontology and archaeology
☐	☒ Animals and other organisms
☒	☐ Human research participants
☒	☐ Clinical data
☒	☐ Dual use research of concern

Methods

n/a	Involved in the study
☒	☐ ChIP-seq
☒	☐ Flow cytometry
☒	☐ MRI-based neuroimaging

Animals and other organisms

Policy information about studies involving animals; ARRIVE guidelines recommended for reporting animal research

Laboratory animals	Long-Evans rats, Male, age 30-40 weeks
Wild animals	No wild animals were used in thise study.
Field-collected samples	No field-collected samples were used in this study.
Ethics oversight	Regierungspräsidium Freiburg, Germany, Abteilung Landwirtschaft, LändlicherRaum, Veterinär- und Lebensmittelwesen

Note that full information on the approval of the study protocol must also be provided in the manuscript.
